# Improving the response of primary care providers to rural First Nation women who experience intimate partner violence: a qualitative study

**DOI:** 10.1186/s12905-020-01053-y

**Published:** 2020-09-21

**Authors:** Kristin Rizkalla, Marion Maar, Roger Pilon, Lorrilee McGregor, Maurianne Reade

**Affiliations:** 1 Department of Interdisciplinary Health, School of Rural and Northern Health, Laurentian University, 935 Ramsey Lake Road, Sudbury, ON P3E 2C6 Canada; 2 Division of Human Sciences, Northern Ontario School of Medicine, 935 Ramsey Lake Road, Sudbury, ON P3E 2C6 Canada; 3 School of Nursing- Department of Health, Laurentian University, 935 Ramsey Lake Road, Sudbury, ON P3E 2C6 Canada; 4 Division of Clinical Sciences, Northern Ontario School of Medicine, 935 Ramsey Lake Road, Sudbury, ON P3E 2C6 Canada

**Keywords:** Northern Ontario, Indigenous women, Intimate partner violence, Primary care providers

## Abstract

**Background:**

Some legacies of colonialism are that Indigenous women living in Canada experience higher rates of intimate partner violence (IPV) and that violence is often more severe relative to non-Indigenous women. This results in avoidable physical, psychological, emotional, financial, sexual and spiritual harm in the lives of Indigenous women, families, and communities. Trusted primary care providers are well positioned to provide brief interventions and referrals to treatment and services, but little is known about the providers’ preparedness to support Indigenous women. Information on what enables or prevents providers to respond to Indigenous patients who experience IPV is needed in order to ensure this potential lifeline for support is realized.

**Methods:**

The purpose of this community-based participatory study was to elucidate the barriers and facilitators to care for rural Indigenous women who experience IPV from the perspectives of primary care providers and to recommend strategies to improve their preparedness. Using a Grounded Theory approach, we conducted qualitative research with 31 providers to discuss their experiences with patients affected by IPV.

**Results:**

The results showed providers often feel a degree of unpreparedness to deal with IPV in a clinical setting.

Underlying the feelings of unpreparedness were:

- Recognition of patients’ under disclosure of IPV due to stigma, shame and fear
- Lack of formal provider training on appropriate approaches to IPV
- Lack of referral network due to fragmented, scarce services for IPV
- Lack of understanding of jurisdictional complexity of First Nations and non-First Nations specific services for IPV
- Uncertainty how to negotiate cultural safety around IPV
- Multiple-role relationship & confidentiality dilemmas characteristic of small communities
- Risk of jeopardizing patient-provider relationship

**Conclusions:**

Our recommendations to improve provider preparedness to address IPV include reducing the stigma of IPV; creating effective referral pathways; improving cultural safety within the referral network; developing services for perpetrators; engaging natural helpers in the community, and; developing policies, procedures and continuing education related to patients who experience IPV in the clinical and community setting. We suggest that increasing providers’ comfort to respond to IPV for rural and Indigenous women will ultimately lead to improved safety and health outcomes.

## Background

*Identifying a problem that you can’t do anything about is not necessarily a wonderful thing.*

{Quote by primary care provider}

Around the globe, intimate partner violence (IPV) is one of the most common forms of gender-based violence. Historically, the terms “domestic/family violence” have been used to describe violence between someone and an intimate member or family member; however, IPV is a more nuanced and encompassing term to describe violence between partners who may not be confined to domestic or familial settings. IPV is defined as physical, psychological, emotional, financial, sexual, or spiritual violence experienced in any intimate relationship and is a grave social issue associated with many adverse health outcomes [1–3]. Although seen among all socio-economic, religious, ethnic, and cultural groups, Indigenous^1^ women in Canada experience elevated rates of IPV, which is best explained within the context of multi-generational trauma experienced by Indigenous communities as a consequence of colonization and forced assimilation [5, 6].

The estimates of the prevalence of IPV experienced by Indigenous women in the existing literature vary and depend on factors such as levels of underreporting of IPV, methodology employed by a study, location of study (e.g.: rural vs. urban area), how researchers defined IPV, and willingness to disclose IPV in household surveys and personal interviews [7, 8]. Thus, reported estimates of IPV experienced by Indigenous women are variable and difficult to ascertain, with studies reporting rates between 25% and 90–100% in some populations [8, 9]^2^. Despite this range in estimates, authors agree unanimously that Indigenous women in Canada face elevated rates of IPV victimization *relative* to non-Indigenous women.

For example, Brownridge [10] conducted a study with two nationally representative populations in 1999 and 2004 and found that Indigenous women were four times as likely to experience IPV compared to non-Indigenous women. In addition, the Ontario Native Women’s Association reports significant rates of IPV: Indigenous women are eight times more likely to experience abuse compared to non-Indigenous women [7]. Furthermore, Indigenous women living in rural and Northern Canadian communities were found to experience higher rates and more severe cases of IPV than those living in urban communities [8].

An integrated framework to explain the origins of gender-based violence from an individual, familial, community and societal perspective is the Ecological Framework [11]. Some individual risk factors that contribute to gender-based violence include witnessing marital abuse as a child and having an absent father; family risk factors include alcohol abuse and male dominance in the family; community risk factors include low socioeconomic status and isolation; and societal risk factors include rigid gender roles and the acceptance of interpersonal violence. However, to understand IPV experienced by Indigenous women, distal causes of these risk factors should be examined through the lens of colonization and the related historical trauma theory. This theory aims to explain how colonial policies has promoted and propagated the use of violence against the Indigenous Peoples, and caused unmeasurable intergenerational grief. Just some of these colonial policies include the forced removal from ancestral lands, and the practice of government enforced removal of Indigenous children from their families for adoption or to attend residential schooling [12]. Historical trauma response is a term to describe the negative affect and behavior associated with multi-generational trauma, such as depression, self-destructive behavior, low self-esteem, suicidal ideation and acts, and restricted emotional expression [13]. Evans-Campbell [12] built on this response into an elaborate framework including familial and community constituents. Inter-generational family level responses to historical trauma includes damaged family communication and parenting stress, while community level responses include the disintegration of traditional culture, internalized racism, widespread alcoholism, and other physical ailments. She asserted that the historical trauma theory is applicable to all Indigenous Peoples who have experienced the colonization [12].

In a clinical setting, primary care providers^3^ are in a unique, frontline position to promote the well-being of those affected by IPV and to offer support to prevent future incidents [14]. These providers interact with patients who are experiencing IPV, either through suspected or direct disclosure of violence. One study suggested that women are not likely to disclose violence in a health care setting unless directly asked [15], but even when patients present with symptoms indicative of IPV, providers were often reluctant to ask about abuse and grapple with a variety of barriers that prevent them from addressing the abuse [16]. Among the studies reviewed, health care providers identified the following barriers: time restraints during the clinical encounter; discomfort in asking; fear of offending; lack of training in how to ask and intervene; cultural or language barriers; attitudes and behaviors from the one experiencing IPV; partner presence; lack of resources/referrals; and a lack of disclosure/denial of abuse. Conversely, there are also factors that could enable providers to ask about abuse and help patients who are experiencing IPV more effectively, such as being an older and more experienced provider, attending training to respond to abuse, as well as having a history with abuse themselves [17].

Research investigating the intersection between the health care system and IPV experienced by rural, remote and Northern Indigenous women is scarce [18]. Most recently, Wuerch and colleagues [18] conducted a qualitative study with community and justice service providers in northern Saskatchewan, Canada, a region whose population is predominantly Indigenous. The aim of their study was to investigate challenges providers faced in meeting the needs of women who experience IPV. They reported that providers perceived that community members held stigma towards mental health services. This stigma may stem from personal notions such as trying to put one’s best foot forward, and structural factors like high employee turnover and a lack of trust patients may have with their provider, which may in turn hinder strong and trusting patient-provider relationships. Providers also expressed that survivors of IPV may be deterred from reporting abuse because perpetrators of IPV in northern communities are often perceived to not be held accountable to the same degree as they might be in urban communities.

Given the many barriers to accessing support services, primary care encounters could represent a lifeline for abused women, especially in low resource environments which are often found in rural Indigenous communities. The partner communities involved in this study identified the lack of a strategic approach to address IPV and the need for a multi-pronged solutions in the services sector (described elsewhere) [19], including practical steps to improve the response to IPV in primary care, especially with First Nations women living on reserve.^4^ To support this need we researched the barriers that primary care providers encounter to support First Nations women who might experience IPV.

## Methods

### Objective

This research was part of a larger initiative conducted by several First Nations communities in collaboration with researchers at the Northern Ontario School of Medicine. The study identified IPV as a significant health and social issue prioritized for community based participatory research. During the planning stage, providers expressed a lack of coordinated approach and an uncertainty in their preparedness to approach this complex phenomenon in the clinical. The overall aim of this research was to identify the perceived barriers and facilitators in the primary care setting to respond to First Nations who experience IPV and to provide recommendations to improve the response to IPV at a clinical and community level.

### Design

We employed a community- based participatory research (CBPR) approach to this study, in which we worked closely alongside community members and organizations throughout each stage of the research process from identification of the research question to knowledge translation [20, 21]. CBPR was used in such a way that this research would be tailored, beneficial and meaningful to the collaborating First Nations and the Family Health Teams on Manitoulin Island.

### Setting

This study took place on Manitoulin Island, in North Eastern Ontario. The Manitoulin District has a population of over 13,000, whereby approximately 5260 are Indigenous. Manitoulin District is comprised of more than 10 villages, seven Anishinabe First Nation communities and many hamlets [22]. The First Nations communities receive health service provision from federally funded health centers on reserve, from one provincially funded Aboriginal Health Access Center as well as from providers working in private practice and Family Health Teams off reserve [23]. In this study, providers participated from two First Nations Health centres in two communities and from two Family Health Teams in two towns (whose staff also work in the Emergency Room), located at two hospital sites.

### Ethics approval

This research project obtained ethics approval from the Manitoulin Anishinaabek Research Review Committee and Laurentian University Research Ethics Board.

### Participants

For participant selection we contacted health team leads to ask if their team would be interested in contributing to the study. Posters advertising the nature, relevance and expected outcomes for the study were relayed to the health team leads by email. For the purposes of this study, an eligible primary care provider was defined as any professional in the healthcare system who provides direct patient or client services and is accessible through self-referral. In addition, these individuals should have had prior experience with IPV in their practice or find IPV relevant to their practice. Eligible providers included regulated and non-regulated health providers, such as physicians, registered nurses, nurse practitioners, mental health workers, physiotherapists, social workers and community health workers.

### Data collection

The participants were invited to share, over lunch, interactions with individuals in clinic, home care, hospital inpatient, emergency room settings and social situations. Before the start of a focus group or interview, an information page of the study was given for review by participants and consent was given to record the session. The lead and senior author collaboratively conducted the facilitation of interviews and focus groups (see Appendix 1). The senior author is a qualitative researcher with more than 20 years of experience in First Nations and rural health research. Each focus group lasted on average 2 h, while semi structured interviews lasted on average 1 h. The participants who were not able to attend a focus group or who had been suggested as a beneficial resource by focus group participants were invited to participate in a semi-structured interview.

Consistent with Grounded Theory we applied theoretical sampling to test emergent observations and theories and to better understand nuances and contradictory experiences between the providers. A total of four focus groups and two in-depth semi-structured interviews with healthcare providers were conducted, at which point data saturation was reached.

### Data analysis

The analysis of data followed a Grounded Theory approach as outlined by Kathy Charmaz [24], whereby data collection and data analysis were performed concurrently. The Grounded Theory approach to analysis is inductive, in that themes and categories emerge directly from the data and were not pre-determined [25]. The data was transcribed verbatim and the initial coding of the data was done by two researchers (KR, MM) using qualitative software, NVivo 12. Data was scanned line by line, and then the researchers moved on to grouping these words, ideas or phrases into larger categories, or themes [24]. To ensure the validity of emerging themes and categories, coding of the data was performed independently by the co-researchers and then compared, and consensus was reached by involving all researchers in the discussion. Emerging themes were discussed after each data collection session between the primary and senior researcher. A core category that defined the main patterns of concern was established. Member checking was conducted by involving one provider and one community member in the analysis as well as presenting results back to the community for discussion and feedback.

## Results

Thirty-one participants contributed in either a focus group or semi-structured interview. Two of the focus groups as well as the semi-structured interviews were held with members of Family Health Teams in mainstream organizations, while the remaining two focus groups were held at First Nations health organizations. Of the 31 participants, six (19.4%) self-identified as physicians, five as social workers/personal social workers (16.1%), two as registered nurses (6.5%), 13 as community health workers^5^ (41.9%), and five as other primary care workers (16.1%). “Other” primary care workers can be very specific positions to Family Health Teams or First Nation health teams, therefore the grouping is used to protect participants’ identities. Examples of these positions include physiotherapists and administrative workers. The majority of participants were female (90%, *n* = 28) and the rest were male (10%, *n* = 3) and approximately half of the participants were Indigenous.

### Grounded theory results

**Fig. 1:**
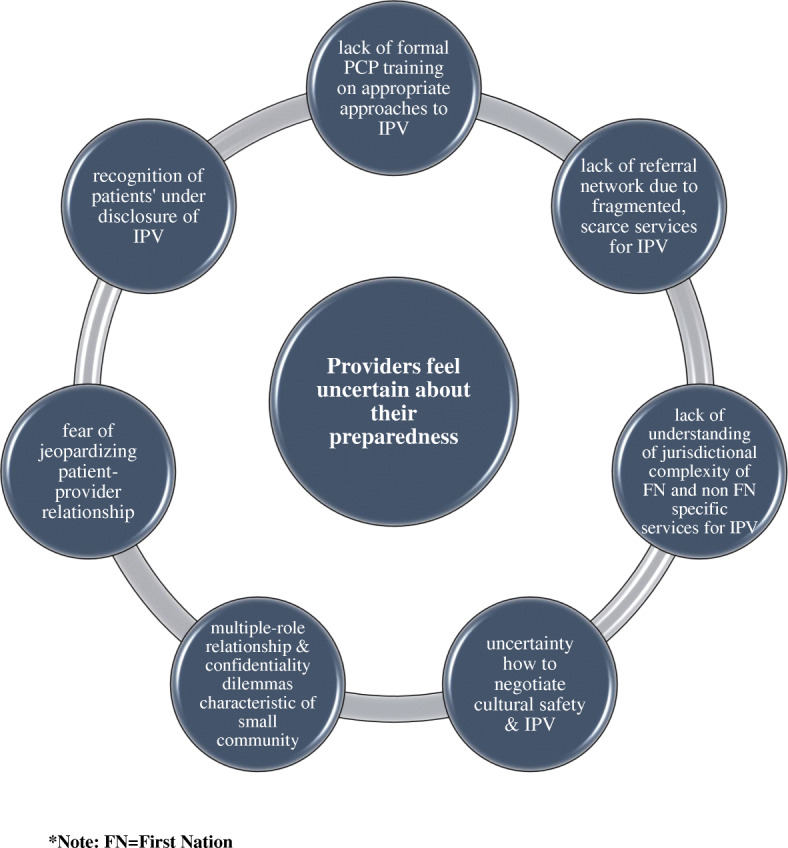
Model of the multiple factors that contribute to providers feeling a lack of preparedness

A core category of concern held by providers when they respond to women who experience IPV was discovered during Grounded Theory analysis. The core concern was the uncertainty of adequate preparedness to respond to women who experience IPV in the primary care setting, compounded by various barriers (see Fig. 1). In turn, this concern can lead to a suboptimal approach or lack of response to IPV by providers**.**

We identified seven main barriers that contribute to the core phenomenon of the unpreparedness to consistently respond to IPV.

1. Recognition of patient under disclosure of IPV due to stigma and shame
2. Lack of formal provider training on appropriate approaches to IPV
3. Lack of understanding of jurisdictional complexity of First Nations and non-First Nations
4. Uncertainty how to negotiate cultural safety & IPV
5. Multiple-role relationship and confidentiality dilemmas characteristic of small communities
6. Fear of jeopardizing patient-provider relationship
7. Lack of referral network due to fragmented services and limited access to these services for IPV

Description of Barriers that contribute to lack of preparedness.

### Recognition of patients’ under disclosure of IPV due to fear, stigma and shame

Many providers felt what hindered them from helping both First Nations patients experiencing IPV was women’s reluctance to disclose. Providers saw patient fear, shame or stigma as one element making it difficult to identify and respond effectively to IPV.

*When you look at intimate partner violence, there’s a lot that’s not reported in our communities. It’s well hidden.*

{Community 2, Participant 5}

*There's often times reluctance to disclose even if you ask several times.*^6^

{Community 1, Participant 1}

Sometimes, even when resources are available, patients will not access the services because of fear, stigma or shame. One reason for not disclosing is the shame over staying in the relationship or fear of being pressured into leaving the relationship by providers.

*The women's shelter on the island does offer outreach services … it does home visiting as long as it's safe to do home visiting. … All of those things are accessible, but still I find people reluctant to be connected to the women's shelter with the perception that they'd have to leave their relationship.*

{Community 1, Participant 2}

Another fear that especially affects First Nation mothers is rooted in the historic high rates of child apprehensions and while in the child welfare system Indigenous children in Canada have suffered high rates of neglect, abuse and even death [27]. Since disclosing IPV might lead to child apprehension, the mother may feel that it is a greater risk than remaining in a home with violence.

*I think that there’s a reluctance to report to us, because there’s an understanding out there of what we need to report to [Children’s Aid Societies].*

{Community 2}

Awareness of reporting requirements may cause sufficient fear to prevent any disclosure for First Nations mothers.

### Lack of formal provider training on appropriate approaches to IPV

Many providers expressed that a lack of training prevented them from addressing the complex issue of IPV in clinical encounters.

*You can't open a can of worms because you don't have training for that.*

{Community 1, Participant 1 referring to Participant 2}

Moreover, dealing with IPV was described as potentially precarious by many providers.

*Well we do want to address violence, especially sexual violence, the topic that has been at our table for a year and a half, two years, maybe longer. And then I think part of the challenge is how we’re going to do it, because we know once we open it, that the potential for something to just explode out is high. So we want to be sure we’re experienced enough to handle it.*

{Community 3, Participant 9}

Coupled with the perception of high risk, there was confusion surrounding protocol or procedures regarding IPV in a clinical setting and perceived differences between professions. Some expressed concern that there was no explicit response to IPV at all within their discipline:

*There's really no standard in our profession on how you deal with [IPV], it's kind of a personal thing, or you call the practice advisor and hope you get somebody who might give you a different answer than someone else.*

{Community 1, Participant 2}

A physician noted that although they had learned about IPV in postgraduate education, they were not well prepared to deal with it on a practical level:

*It's addressed in residency, but … at the end, … you are sort of looking out for it, you know how to identify it, but then again, what do you do with it?*

{Community 1}

Non-physician providers discussed not having learned about IPV in their profession and that presented as a barrier to respond properly. One physiotherapist noted:

*They didn't really touch on this kind of stuff [IPV education] in physio school as much.*

{Community 1, Participant 2}

Furthermore, IPV within a First Nations context represents a particular gap in health care provider curriculum. A social worker spoke about overall gaps in awareness of First Nations culture, history of colonization or ways of knowing within primary care as a further obstacle:

*[There is] not nearly enough [ … ] But bare minimum, I think anybody that wants to work with First Nations populations should at least take that [First Nations cultural competency course], or take something similar to that. So that they have some context, because if you have absolutely no context and you think it’s all pow-wows and jingle dresses...*

{Community 1}

The reference to pow-wows underscores the understanding by this provider that a simplistic level of cultural understanding can be observed among many providers.

The lack of a coordinated application of a valid screening tool in their practice resulted in many providers being unsure how to broach the topic of IPV with patients:

*I'm not sure what the 2018 best practice is: screen, don't screen? Or secondary screening only for suspicious behavior or something?*

{Community 1, Participant 3}

Providers also wondered whether one screening method was more valid than another, and whether more direct or indirect questions would be more effective in opening up a dialogue on IPV, and if opening dialogue was actually helping patients or not.

*You don't know how often I ask the wrong question, like … (what) would be THE best question to elicit the highest or to pick up the biggest number of cases?*

{Community 2, Participant 1}

One provider summarized the dilemma for providers due to the lack of good information about IPV:

*So there’s that whole mystery, it would be nice if people found out what they can do and what options are there if they’re hearing or witnessing this. So I don’t think that’s very well known. People are scared to make that call, or people are scared on how to support. So I think learning more of that would be beneficial*.

{Community 3, Participant 6}

One provider reflected how the ambiguity lead to her feeling powerlessness in her role as a primary care provider, especially upon disclosure of intimate partner violence:

*[Intimate partner violence] is not even reportable. It's not reportable in a nursing home. It's not part of the process... So, you sort of appreciate them for opening up, encouraging them to maybe take it further or get some sort of counselling. But it’s sort of an unsatisfying thing, because how many times would you let an abused woman out of your emerg?*

*{Community 1, Participant 1}*

### Lack of understanding of jurisdictional complexity of First nations and non-First nations specific services for IPV

A few physicians expressed how the siloed funding imposed on First Nations and the rural health and social services system interfere with the effective delivery of care towards patients who experience IPV,

*So she was a Wiikwemkoong patient, but I had referred her to M‘Chigeeng [services]. And they all have very long names that I can't pronounce yet, so it is a bit overwhelming for me.*

{Community 2, Participant 3}

The comment implies that the patient as a member from one community was ineligible for services provided in another community, however Family Health Team staff often do not understand the First Nations health care system. Regional providers have similar restrictions which fragments care and impedes access.

*If we're having as health care professionals difficulty navigating the system and figuring out where people get the help, how much more so for actual patients in the community? [ … ] I have to admit to not knowing specifically what resources would be available other than the general mental health services in each of the First Nations communities.*

{Community 2, Participant 1}

### Uncertainty how to negotiate cultural safety and IPV

Another concern that providers voiced about responding to First Nations women who may be experiencing IPV was a fear of being perceived as culturally inappropriate, judgemental or being labeled as an “*outsider, interfering in this relationship.”* {Community 1, Participant 5}. Similarly, First Nations providers also acknowledged cultural safety concerns when it came to connecting with First Nations women who experience IPV with counselling:

*Counselling, especially if it’s non-Indigenous providers coming in, they’re very Westernized in terms of, like some of our young people don’t want to sit in the office awkwardly with somebody to try and figure out what’s going on with them. They don’t talk about ever doing other activities, like taking a walk, doing things that way, or any activity on the land. Because once you get to that point with somebody, it’s just a conversation, and an entire counselling session can happen right there, doing that activity and getting to know one another. So that’s one of the barriers I saw, is that they don’t want to come to the health centre to sit in a room, awkwardly sit there with somebody, trying to explore what’s going on with me, you know?*

{Community 3, Participant 3}

### Multiple-role relationship & confidentiality dilemmas characteristic of small community

Multi-role relationships in small rural communities posed a dilemma with respect to confidentiality. One health care worker commented that even when IPV is suspected based on her observations in the community when she was off duty, there was still a perception of a duty to maintain confidentiality. This in turn limits what some providers feel they are able to do.

*We had a client who had been sexually assaulted and had physical injuries as a result of that violence, and came in, would talk to the female staff … about her injuries. She’d never say how she’d got them, although we heard that she got them. You’re walking on a tightrope of what you could say and what you couldn’t say based on what you knew {through observations in the community}. And for her to finally reveal her story and to finally agree, it was over a month in before she agreed to see a health professional [ … ] But that was a real challenge … to manage her confidentiality, but you wanted to help her and allow her to go through that process, and have her process everything.*

{Community 3, Participant 9}

Often providers find it difficult to balance confidentiality and personal relationships in these situations especially when IPV was disclosed to them personally outside of their conventional working hours.

*What I’ve noticed or experienced [in terms of patients’ IPV], it’s always after hours. Or it’s just friend to friend. [ … ] But where do I draw the line, as a health care professional? I work 8-4. My friendship starts from 4:01 to 7:59 the next day. Am I obligated, because I’m a health care professional, to report this? Or do I do it in confidence as a friend?*

{Community 4, Participant 2}

Patients on the other hand can also struggle with their personal relationships with a primary care provider:

*And people often times don't want to let anything out because they know that it's gonna be, like, their cousin who works as a nurse in emerg, you know, somebody was passing by because they're here for different reasons and it's gonna be known and it all becomes known. So they wanna hide it. They don't want to disclose it to you, to healthcare professionals.*

{Community 2, Participant 1}

Another provider spoke about that it can be prohibitive to seek support for IPV if a relative is working in the services system. Privacy was seen as a big concern to access services.

*[It’s about] recognizing though that a lot of our people are private, and it comes down to confidentiality. That's why we don't see them in the health system too often.*

{Community 2, Participant 5}

### Fear of jeopardizing the patient-provider relationship

Providers also identified their concerns with legal implications for their patients associated with acting on IPV. It is the provider’s duty to report to children’s protective services in the case of suspected IPV that involves children under the age of 16 in the home. One physician noted:

*We always try to tease out whether children are at risk, because children at risk gives you sort of a way to report it, but that might actually ruin your relationship with the patient.*

{Community 1, Participant 1}

In addition, providers may be targeted with criticism for taking action to helping women. Community or family members, who are not yet ready to deal with the issue of IPV especially where physical acts of violence may involve arrests; one family member for example approached a provider asking *“why would you criminalize my father’s behavior?”* {Community 1, Participant 1}.

### Lack of referral network due to fragmented, scarce services for IPV

Some providers mentioned that directing patients who may be experiencing violence in their lives to appropriate agencies in a fragmented rural services landscape is daunting and even overwhelming as described by this participant.

*Wow, you feel... overwhelmed, and that they will share that with you, so it's kind of this sign of just, respect on their part, and a burden at the same time because now what do you with some of that information? Some of that is difficult to know, you know, whether you need to involve the law.*

{Community 1, Participant 7}

What to do with the information once IPV is disclosed was a major concern for medical staff and for social/mental health providers. Details about referrals and resources were frequently unclear, especially the availability of shelter and support services, long wait times, and if those services were culturally safe and accessible to both First Nations and non-First Nations women. Many providers struggled with connecting First Nations patients with the complicated assortment of federally-funded, often community-specific or regional services as well as mainstream provincial services. Some spoke of making inappropriate referrals to First Nations services to First Nations women who were not eligible for these services due to, for example, place of residency. Those who have worked longer in the area helped when possible to connect patients informally:

*I would case manage the linkage into the appropriate community-based agency, because it is a complex system …*

{Community 2, Participant 2}

One physician noted that a lack of resources or challenge in accessing existing resources for First Nations as well as non-First Nations women may prevent a provider altogether from helping a patient who was experiencing IPV.

*Where the rural challenge is greater is the lack of resources.*

{Community 1, Participant 3}

Description of Facilitators to care towards Indigenous women who experience violence.

After discussing barriers, providers shared what they saw as facilitators for responding to IPV in the PC setting as well as the strengths of their patients in improving their own situation.

Perhaps one of the strongest facilitators discussed amongst the sample of providers was the concept of working together as a team across health disciplines. One social worker explains this teamwork well, by saying:

*We try within our clinic to use the multidisciplinary model and to say ‘okay well, [medical doctors] may not have time to ask more about this, on this day, but would you be willing to talk to this person or this person or this person?’*

{Community 2, Participant 7}

Having a social worker embedded in the family health team opens up new opportunity for more comprehensive services as physician appointments may be too short to address many social issues.

*I struggle with how short a doctor’s appointment is. I mean doctors are good people, they would like to ask all the questions, but they’re not in the position to be able to ask all of the questions that would be helpful sometimes. And so I think that pressure to see this many patients … I don’t even think that’s within OUR service delivery kind of training.*

{Community 2, Participant 7}

Sometimes, a team member who has seemingly little impact on helping a patient with IPV may have great insight into the lives of exactly those patients as this physician explained:

*But they're the eyes of the community, right? We don't go in the houses, they do! They can come in the house and they sort of - they provide very interesting insights on our rounds because he's the one who actually goes in the house …*

{Community 1, Participant 1}

### Culturally safe care

Several providers mentioned understanding Indigenous culture and values was an enabler to care involving sensitive issues within a clinical setting and that there were some services that are culturally safe.

*I think knowing a bit about the culture and knowing how the interconnectedness of the First Nations value system, plays a key role in how far you’re going to be able to get. [ … ] When … Betty is telling you that Charlie is abusing her, it’s not just Betty in the room. Betty brings all of her [family] and ancestors with her as well, and being cognisant of that, being understanding of that, being aware of it, being respectful and mindful of that to allow her to talk about how this is impacting her in this holistic way, in this bigger way.*

{Community 1, Participant 8}

On the other hand, Indigenous providers found that culturally sensitive care also entailed giving patients the option to choose cultural options that fit their life path and again this awareness was often present.

*We do have people that practice different cultural traditions and religion. Like we have people who still have very strong ties to our church and then we have people who do ceremonies, but we have different types of ceremonies in our communities too, so when you’re bringing one culture in too, it might not resonate with everyone in our community.*

{Community 4, Participant 3}

Some providers offered what has worked for them practically in terms of screening, which includes using progressive screening, a customized script and other means of flagging IPV.

*I think everyone that works in the health profession should have a script that they feel comfortable with. And to me, it doesn’t matter to me who you are. [ … ] So it may be a combination of both being direct, and also being gentle. So how does that feel for you? Because for me, being authentic, being present, with you right now, is the primary goal.*

{Community 1, Participant 8}

Others mentioned that they were actively trying to identify screening that would work in their practice.

## Discussion

### Barriers

In general, barriers to support women who experience IPV were discussed by providers much more frequently relative to facilitators. This is a result of the complex factors that lead this pervasive level of uncertainty related to responding to IPV, which the providers saw at a survivor, systemic, and provider level. With respect to survivor barriers, the colonial systems and policies that have resulted in historical trauma such as the high rates of violence perpetrated against Indigenous women still operates and creates fear in Indigenous women from further structural violence such as removal of children, due to the violence they are experiencing at home. This creates a dilemma for the women of fear of losing children and shame of their living situation while primary care providers are in a dilemma of addressing the issue and being bound by mandatory reporting that will lead to losing trust. With respect to provider barriers, challenges regarding confidentiality and reluctance to go to their service provider who may also happen to be a family member compound this dilemma. Some of the barriers to address IPV in primary care were similar to those experienced in the justice sector in other rural and Northern communities in Canada and are connected to limited resources or access to resources in rural areas. However, the First Nations health care systems presents with unique barriers given the jurisdictional gaps between First Nations and mainstream health care. With respect to systemic barriers, some primary care providers may experience confusion when faced with the complex health and social services system, especially if they are not knowledgeable about the to the jurisdictional divides that cause gaps in First Nations health and social services. In addition, a clear presenting barrier is a lack in coordinated protocols or procedures regarding intimate partner violence across the health care systems. Currently there is no universal screening; some using screening only once a strong trusting relationship between the patient and provider is established. Clearly, provider training and more supportive services are needed to allow women more choices for safety are urgently needed.

### Facilitators

Previous literature has demonstrated that adequate time to build a relationship with the patient may help providers identify and support patients who are experiencing IPV. This was validated in our study, in which inadequate time was relevant to physicians more so than other disciplines. Social workers, who are often much less restricted in time, acknowledged that a lack of time was a limiting factor for physicians in potentially helping an abused patient. The social workers in this study recommended interdisciplinary collaboration as a way to address this. This teamwork may help to better identify IPV by providing more opportunities to screen for IPV or for case-finding IPV in patients who may not have regular appointments with a physician. Analysis showed that providers valued knowledge of Indigenous values and the ability to offer safe spaces for traditional practices, in order to provide culturally sensitive care. Providers should understand that Indigenous Peoples are diverse and have unique needs. In this sense, culturally sensitive care entails a patient-centered approach, wherein the patient chooses what approach best suits them.

### Recommendations

Our analysis provides the basis for the following recommendations to improve the ability of providers to respond to women who experience IPV in rural and First Nations communities.

#### Continuing to address stigma through community awareness

Providers felt that empowering the patients directly through ongoing public health campaigns is a helpful strategy. Ongoing funding for these campaigns helps patients to better understand the various dimensions of IPV, what acceptable behaviour in an intimate relationship looks like, to reflect upon their experiences and eventually to be able to seek out and access services they need directly. Information pamphlets, educational videos to play in waiting areas and culturally specific posters, particularly if trying to address Indigenous health, are recommended awareness strategies.

#### Create effective referral pathways

A local task force to create appropriate referral pathways should be formed. These pathways could be promoted through information sessions on the locally available services and resources, including access to workshops, counselling and shelter services for rural and First Nations women. All women’s and children’s services and agencies in the area as well as the process for accessing these services or for referring clients should be detailed in writing. This should include criteria who can access services due to catchment area, First Nations status, geographic location as well as cultural safety. This list of resources should also be accessible by patients, especially in the emergency room.

#### Improve cultural safety within the referral network

The idea of cultural safety refers ultimately to supportive, non-judgemental care that suits a patient’s specific needs and is not assumed. It may include offering a First Nation person access to traditional healing such as smudging, sweat lodges and other ceremonies, while understanding that some may select mainstream services. Cultural safety training will be beneficial to increase providers’ ability to approach care and referrals for Indigenous women [28].

#### Develop services for perpetrators

Numerous providers mentioned that many perpetrators were struggling with mental wellness, anxiety and control issues and did not have the skills to change their attitude or behaviours towards women. While providers did not deny that there may be need for involving the justice system in some cases, it was felt that *restorative justice* and rehabilitative programs were seen as key to reducing IPV. Some of the providers from counselling professions explained that the perpetrator have themselves abuse histories. Cultural programs for Indigenous men were suggested, for example “I am a Kind Man” offered in some of the local First Nations health organizations [29].

#### Engage natural helpers in the community

Engaging the natural helpers, knowledge holders, grandmothers, grandfathers, and elders within the communities with provider teams and other service organizations to help survivors and abusers was also seen as long-term strategy to address IPV. Several providers mentioned the resilience demonstrated by the First Nations communities on Manitoulin Island, namely around the fact that there are strong ties within small communities. Therefore, implementing concepts into First Nation programming such as individual and community resilience as well as the seven grandfather teachings were recommended by some First Nation providers. Ungar’s [30] international study explains how resilience is inextricably related to context and culture and delineates three protective processes for resilience: how environmental level variables can be more influential than individual level variables; how facilitative environments can positively impact how individuals, families and communities perceive, navigate and access resources; and how a greater exposure to risk can be mitigated when there are resources that target those specific risks.

#### Develop policies, procedures and continuing education related to patients who experience IPV

Finally, while education and training may differ based on the discipline of a provider, this study has implications for policies, procedures and the education of providers. For example, web-based training on how to speak to patients in a clinical setting may be beneficial in creating a safe and non-judgemental environment for a patient to disclose and seek help for intimate partner violence [31].

To continue, there appears to be a lack of coordinated approach to screening in the health care system on Manitoulin Island, which does not seem unusual given the debate as to whether universal screening (screening regardless of perceived risk) or non-universal screening is most effective [32]. Moreover, the screening protocols as they stand also appear to undermine provider confidence, as they may not be aware of which questions to ask or how to proceed upon disclosure of IPV [33]. To overcome these challenges, a universal and routine screening protocols with clear guidelines ought to be considered. Successful programs were shown to have standardized and direct screening IPV questions, as well as provide protocols on how to assess patient safety, and refer to appropriate services. These guidelines should be disseminated to staff through initial and ongoing mandatory training sessions to increase provider comfort and self-efficacy.

Finally, as this study highlighted the importance of cultural sensitivity in delivery of care, this study may lead to reforms to provider undergraduate training or continuing professional development by educating on First Nations ways of life.

### Strengths & limitations of study

The participants included in this study are from a single district, therefore the results may not be generalizable to other rural, remote or northern districts of Canada. In addition, 90% of the providers involved in this study were women and the perspectives of male providers were not explored in as much detail. One notable strength of this study was the variety of First Nations community staff and conventional health providers and disciplines involved. Based on this broad spectrum of perspectives, we believe our results will resonate with providers who care for Indigenous women elsewhere. Further studies should build on this with direct research with Indigenous women who have experienced IPV.

## Conclusion

Intimate partner violence is an extremely serious health and social issue that affects Indigenous women at elevated rates [5]. This study identified barriers that need to be overcome to alleviate feelings of unpreparedness experienced by primary care providers in supporting Indigenous women who experience IPV. In addition, this study identified facilitators that may help mitigate feelings of unpreparedness. Strategies to ameliorate current conditions should go beyond provider training and include community wide interventions to address stigma, cultural safety and community awareness of healthy relationships. Services for perpetrators are also urgently needed. This study can serve as a starting point to more effectively address IPV in rural and First Nations health care systems and inform future programming in the health care system, as well as inform clinical approaches to intimate partner violence.

## Data Availability

The datasets used and analyzed during this current study are not publicly available due to sensitive nature of the data and for the potential for identifying research participants but are available from the corresponding author on reasonable request.

## Appendix 1

1. Can you tell me, based on your work with your patients/clients, your thoughts on intimate partner violence and how it impacts primary care? How does IPV affect patients/clients (trauma, ability to take care of their health, chronic illnesses, other SDOH, other?) Does it affect First Nations women differently than non-FN? Does it affect First Nation women more than First Nation men?
2. How does IPV come up in interactions with patients? What do you believe is the best screening process? (Probes: Do patients feel safe to disclose? Are they trying to hide it? Does IPV come up often? Do you suspect it often? Do you bring it up with your patients/clients and how? What is the response? Indignation, shame, denial?
3. Is a close relationship with a First Nation patient a barrier or an enabler for delivering care?
4. What resources are available to help patients who are struggling with IPV? Which ones do you regularly refer clients to? Are there differences in services for FN vs non-FN? Do you provide any counselling yourself? (what referrals would you make, what are some key ways to improve IPV related issues in the community?)
5. How prepared do you feel in helping patients experiencing IPV? How about specifically to FN women? Did your training prepare you to support patients with IPV? (Undergraduate and CME).
6. What resources or training are necessary to more effectively deal with IPV? How about specifically to FN women? What would you like to see in terms of training? Is there training specific to FN people that you would need?
7. What would you like to see in order to improve responses to IPV from the primary care perspective?
8. Imagine we spoke again about this topic in 5 years. At that time you are
  happy to describe to me that there have been some very positive changes
  related to how IPV is addressed in the health and social services system.
  What positive changes would you hope to report?
9. Will any of the information discussed today CHANGE your future practice?

### Probes

1. What do you mean by that?
2. Has anyone else had the same experience?
3. Do you think other primary care providers have had similar experiences?
4. Has anyone else had a different experience?
5. Are you able to provide some examples?
6. I would like to hear more.
7. Please expand on that thought

## Abbreviations

IPV: Intimate partner violence
CBPR: Community based participatory research

## Footnotes

1 Unless stated as “First Nation”, the term “Indigenous” is an all-encompassing term which can refer to Peoples who identify as First Nation (status and non-status), Métis or Inuit [4]

2 The statistics reported, unless otherwise stated, do not represent First Nation communities living on reserve.Most databases from National surveys report data with “self-identified Aboriginal People” and almost always exclude First Nation communities on reserve [9].

3 Hereafter referred to as provider/s

4 In Canada, a reserve is area of land set apart for use by a First Nations community.

5 Community health workers are an under-recognized and unregulated group of individuals who provide health-related services for fellow community members. Examples of titles of community health workers depend on geographic area and populations served, some of which include Community Health Workers, Community Nutrition workers, and Community health representatives [26].

6 Note: The language patterns in the quotes are slightly modified to help protect the anonymity of the providers on Manitoulin Island.
